# IT-Führungsrollen des Top-Management-Teams im öffentlichen Sektor

**DOI:** 10.1365/s40702-022-00873-w

**Published:** 2022-04-28

**Authors:** Andreas Paech, Dominik Vogel

**Affiliations:** Fakultät für Wirtschafts- und Sozialwissenschaften, Universität Hamburg, Hamburg, Deutschland

**Keywords:** Job Mining, IT-Führungsrollen, Kompetenzen, Verantwortungen, Aufgaben (KVA), Öffentlicher Sektor: CTO–CDO-Dyade, Job Mining, IT-Leadership Roles, Competencies, Responsibilities, Tasks (CRT), Public Sector, CTO–CDO Dyad

## Abstract

Mittlerweile gibt es auch in Organisationen des öffentlichen Sektors häufig mehrere IT-Führungsrollen im Top-Management-Team (TMT). Wir wissen bisher aber sehr wenig darüber, wie diese IT-Führungsrollen ausgestaltet sind und wie sie sich voneinander abgrenzen lassen. Dieser Artikel nutzt Job Mining von Stellenausschreibungen im öffentlichen Sektor, um mittels einer qualitativen Inhaltsanalyse Kompetenzen, Verantwortungen und Aufgaben der IT-Führungsrollen zu identifizieren und Handlungsempfehlungen zu formulieren. Während zu den geforderten Kompetenzen von IT-Führungsrollen u. a. ein abgeschlossenes Hochschulstudium, Sprachkenntnisse sowie fach- und verwaltungsbezogene Kenntnisse zählen, zeigen die Verantwortungen verschiedene Ausprägungen. Die Aufgaben sind rollenspezifisch formuliert, zeigen jedoch eine Reihe von Überschneidungen zwischen den IT-Führungsrollen. Diese Überschneidungen legen eine CTO–CDO-Dyade mit übergeordneter CIO-Rolle nahe. Die praktisch ausgerichteten Handlungsempfehlungen umfassen u. a. eine höhere Gewichtung von Englisch- und Digital-Kenntnissen sowie lebenslangem Lernen.

## Abstract (en)

In the meantime, even in public sector organizations, there are often several IT leadership roles in top management. So far, however, we know very little about how these IT leadership roles are designed and how they can be distinguished from one another. This article uses Job mining of job advertisements in the public sector in order to identify competencies, responsibilities and tasks of the IT management roles by means of a qualitative content analysis and to formulate recommendations for action. While the competencies required for IT management roles include a university degree, language skills, and specialist and administrative knowledge, the responsibilities show different characteristics. The tasks are formulated in a role-specific manner, but show a number of overlaps between the IT management roles. These overlaps suggest a CTO–CDO dyad with an overarching CIO role. The practical recommendations include a higher weighting of English and digital skills as well as lifelong learning.

## Einleitung

Der öffentliche Sektor steht vor der großen Herausforderung, sich umfassend zu digitalisieren, eine über Jahrzehnte gewachsene IT-Landschaft zu konsolidieren und den Betrieb und die Fortentwicklung zahlreicher kritischer IT-Systeme zu gewährleisten. Wie zentral diese Herausforderung für die gesamte Gesellschaft ist, hat uns die COVID-19-Pandemie gezeigt. Um diese Aufgaben zu bewältigen hat der öffentliche Sektor in den vergangenen Jahren nicht nur zunehmend IT-Personal eingestellt, sondern auch IT-Führungsrollen im Top-Management-Team (TMT) geschaffen. Dies umfasst Chief Digital Officers (CDO) und Chief Information Officers (CIOs), aber auch TMT-Positionen für Technologie (CTO), Produkte (CPO^1^) und Informationssicherheit (CISO).

Diese Entwicklung ist durchaus bemerkenswert, da sie für die etablierten Strukturen des öffentlichen Sektors untypisch ist. In diesen Strukturen sind Top-Führungskräfte üblicherweise für streng abgegrenzte Aufgabenbereiche verantwortlich. Führungsrollen mit aufgabenübergreifenden Verantwortlichkeiten, wie sie bei IT-Führungsrollen im TMT üblich sind, sind in dieser Logik traditionell nicht vorgesehen.

Umso erstaunlicher ist, dass wir bisher kaum etwas über diese IT-Führungsrollen im TMT des öffentlichen Sektors wissen. Hierzu gehört auch, dass weitgehend unklar ist, wie die einzelnen Rollen voneinander abgegrenzt werden oder wie sie ausgestaltet sind. Dieser Artikel greift diese Forschungslücke auf und nutzt eine qualitative Analyse von öffentlichen Stellenanzeigen, um einen ersten Einblick in Ausgestaltung und Abgrenzung von IT-Führungsrollen im TMT des öffentlichen Sektors zu gewinnen. Stellenanzeigen sind in diesem Kontext von besonderem Interesse, da sie verbindlichen Auswahlkriterien zur Bestenauslese festlegen und insofern mit großer Sorgfalt erstellt werden müssen.

Dieser Artikel nutzt die Stellenanzeigen, um folgende Forschungsfrage zu beantworten: Welche Kompetenzen, Verantwortungen und Aufgaben (KVAs) werden den einzelnen IT-Führungsrollen zugewiesen und wie lassen sich die verschiedenen Rollen darauf aufbauend voneinander abgrenzen? Der Artikel vergleicht somit theoretische Annahmen über IT-Führungsrollen im TMT mit der empirischen Realität des öffentlichen Sektors und leitet darüber hinaus Handlungsempfehlungen zur Optimierung der Stellenausschreibungen sowie zukünftigen Forschungsbedarf ab.

## Datenerhebung

**Abb. 1:**
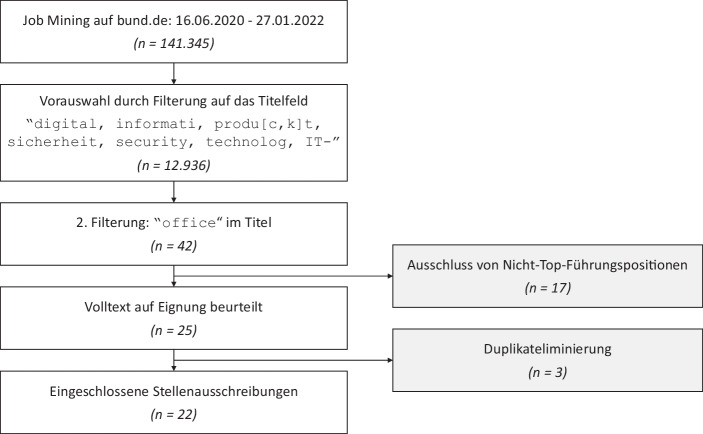
Datenerhebung

Job Mining ist ein etabliertes Analyseinstrument im Human Resource Management zur Erstellung von Anforderungsprofilen (Bensberg und Buscher 2016). Es wurde für diese Studie als *Datenerhebung*sinstrument im Zeitraum 16.06.2020–27.01.2022 durch einen Web-Crawler umgesetzt, der alle Stellenangebote auf bund.de täglich erfasst, bereinigt und speichert. bund.de richtet sich an Behörden und Behördendienstleister der Bundes‑, Landes- und Kommunalverwaltung, um Stellenangebote und Ausschreibungen zu veröffentlichen. Auch die für die vorliegende Fragestellung über IT-Führungsrollen prädestinierte Behörde im öffentlichen Dienst, ITZBund (Informationstechnikzentrum Bund), veröffentlicht seine Stellenangebote neben seinem eigenen Jobportal auch auf bund.de. Im genannten Zeitraum wurden insgesamt 141.345 Stellenangebote auf bund.de veröffentlicht (siehe Abb. 1). Davon konnten 12.936 den Fachbereichen der IT-Führungsrollen zugeordnet werden, wobei in der Suchabfrage sowohl englische als auch deutsche Bezeichnungen berücksichtigt wurden. In einer zweiten Vorauswahl folgte eine Einschränkung auf das TMT, dadurch dass „Office“(r) im Titel enthalten sein sollte. Eine Filterung auf „Manage“(r) oder „Leitung“/„Leiter:in“ hätte hingegen auch mittlere Managementrollen eingeschlossen. Nach Ausschluss von Nicht-Führungspositionen (Assistenz- und Sekretariatsstellen) und Duplikaten verblieben 22 Stellenausschreibungen zur qualitativen Datenanalyse.

**Abb. 2:**
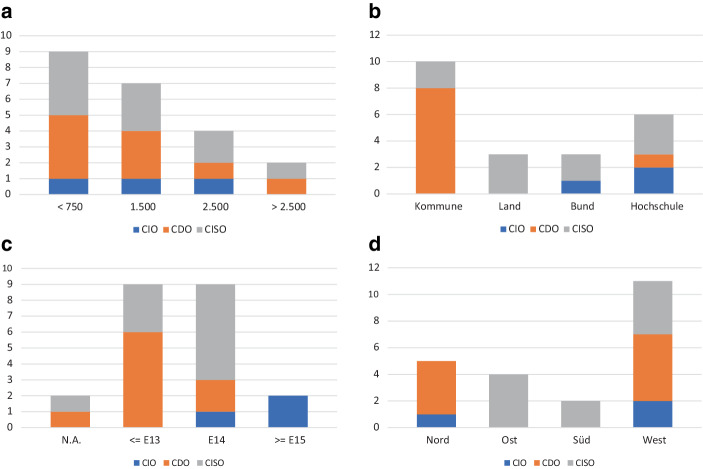
Stichprobencharakteristika der Stellenausschreibungen. **a** Behördengröße, **b** Behördenart, **c** Entgelt, **d** Region

Die 22 eingeschlossenen Stellenausschreibungen verteilen sich auf neun CDO-, zehn CISO- und drei CIO-Stellen. CTO- oder CPO-Stellen sind nicht vertreten. Abb. 2 zeigt die Verteilung der IT-Führungsrollen der ausgewerteten Stellenausschreibungen nach Behördengröße und -art sowie Entgelt und Region.

## Datenauswertung

Die Datenauswertung erfolgt als qualitative Inhaltsanalyse (Mayring 2008). Die Auswertung erfolgt chronologisch nach Veröffentlichungsdatum. Die Kodiereinheit sind einzelne zusammengehörende Wörter bzw. bei Aufzählungen das gesamte Aufzählungsitem. Die Kontexteinheit ist ein einzelner Absatz. Der Kategorienbaum ist auf oberster Ebene deduktiv aus den einleitend genannten Forschungsfragen abgeleitet und gliedert sich in Kompetenzen, Verantwortungen und Aufgaben. Die Kompetenzen werden in Knowledge, Skills, Attributes und Others (KSAOs) unterteilt. Die Aufgaben unterteilen sich in rollenübergreifende Aufgaben sowie rollenspezifische Aufgaben für CDO, CISO und CIO. Unterhalb der Unterkategorien sowie Verantwortungen werden untergeordnete Codes induktiv aus dem Datenmaterial abgeleitet, so dass der Kodierbaum drei Ebenen umfasst: erste Ebene: KVA, dann Unterkategorien für Kompetenzen (KSAOs – Knowledge, Skills, Attributes und Others) und Aufgaben (Rollen – CDO, CISO, CIO) und auf letzter Ebene die aus dem Datenmaterial induktiv abgeleiteten Ergebniscodes. Tab. 1 zeigt ausgewählte kodierte Segmente zugeordnet zu einer Kategorie. Es wird davon ausgegangen, dass es für gleiche Verantwortungen unterschiedliche Ausprägungen gibt, so dass die axiale Kodierungstechnik von Strauss und Corbin (1990) angewendet wird. Insgesamt wurde das Material in drei Iterationen ausgewertet; in der dritten Iteration erfolgte keine Änderung der induktiven Kategorieteilbäume mehr. Der Datenauswertung erfolgte computerunterstützt mit MAXQDA.

**Tab. 1:** Kodierbeispiele

**Kompetenzen**	
Knowledge > Lebenslanges Lernen	*Regelmäßiger Besuch von relevanten Konferenzen, Tagungen und Fortbildungen* *Eigenmotivation und Interesse an regelmäßiger Weiterbildung* *Wir bieten: […] Fortbildungsmöglichkeiten*
Skills > Digital & IT	*Ausgebildetes Grundverständnis im Bereich der Informationstechnik* *Gefestigte Kenntnisse rund um digitale Themen, Trends und Technologien […]* *höchste IT-/Digitalisierungsaffinität*
**Verantwortungen**	
Repräsentation	*Mitwirkung […] in den fachpolitischen Themenbereichen des Ministeriums* *Fachliche Vertretung der Interessen […] in nationalen und internationalen Gremien* *Repräsentation […] nach innen und außen* Ansprechpartner*in für den digitalen Wandel nach außen (Bürger*innen, Ämter, Kommunen, Kreis, Land, Bund, private Firmen, WKS usw.)
Öffentlichkeit/ Partnerschaften	*Vernetzung mit den CISOs der staatlichen Hochschulen des Landes NRW und anderer Einrichtungen* *Proaktive Vernetzung mit den relevanten Akteurinnen und Akteuren* *Abstimmung der jeweiligen Öffentlichkeitsarbeit*
**Aufgaben**	
Chief Digital Officer	*Konzeption, Akquise und Management von Innovationsprojekten* *steuern den digitalen Transformationsprozess* *Digitalisierung als konzeptionelle Querschnittsaufgabe*

## Ergebnisse

### Kompetenzen

In der Regel wird ein abgeschlossenes Studium gefordert, nur selten ist alternativ eine Ausbildung in Verwaltung oder IT und langjährige Erfahrung ausreichend. Die Fachrichtung des Studiums ist nicht relevant, jedoch ist Public Management als mögliche Fachrichtung fast immer genannt, meistens auch Wirtschaftswissenschaften oder (Wirtschafts‑)Informatik. Sozial‑, Geistes- oder Rechtswissenschaften sowie naturwissenschaftliche Fächer werden nur in Einzelfällen genannt. In vier Fällen werden gute allgemeine Englisch-Kenntnisse gefordert, z. B. für die CISO-Stelle im Auswärtigen Amt auf dem Niveau B1 des Europäischen Referenzrahmens. In allen anderen Stellenausschreibungen sind keine Englisch-Kenntnisse gefordert. Konkrete Weiterbildungen und Zertifizierungen werden für CISO-Stellen benötigt (BSI-Grundschutz, ISO 27000, IT-Auditor), jedoch nicht für die anderen IT-Führungsrollen. Darüber hinaus werden (nicht zertifizierte) Kenntnisse in IT- und Vergaberecht, Verwaltungsstrukturen und -prozessen sowie Projekt‑, Change- und Prozessmanagement erwartet. Agile Methoden wurden nur einmal gefordert. Je nach Rolle werden mehr (CIO, CISO) oder weniger (CDO) Kenntnisse in IT-Management und -Governance sowie IT-Architektur gesucht. Ein weites Spektrum findet sich in Bezug auf lebenslanges Lernen in den Stellenausschreibungen: es reicht von der grundsätzlichen Bereitschaft und dem Angebot zur Weiterbildung bis als Aufgabe definierten „regelmäßigen Besuch von relevanten Konferenzen, Tagungen und Fortbildungen“.

Die benötigten Skills werden zum großen Teil rollenunabhängig definiert. So benötigen Bewerber:innen eine „mehrjährige einschlägige“ Berufserfahrung, für CISO-Stellen werden zwei oder drei Jahre als konkrete Untergrenze definiert. Bewerber:innen sollen über eine strategisch-analytische Denkweise und kreative Konzeption verfügen. Die ausgeprägten sozialen und kommunikativen Kompetenzen werden neben der Netzwerkkompetenz stets betont. Bewerber:innen sollen über Führungserfahrung von interdisziplinären Teams verfügen. Die geforderten IT/Digital-Skills erstrecken sich wieder über ein breites Spektrum: es werden mind. Grundverständnis und eine (hohe) Affinität für IT gefordert, durchschnittlich reichen „gute Kenntnisse inkl. MS-Office“ aus. Am oberen Ende des Spektrums finden sich vereinzelt geforderte „gefestigte Fachkenntnisse“.

Von den Bewerber:innen werden die Attribute Innovations- und Gestaltungswille, selbstsicheres Auftreten und offener Umgang mit Menschen sowie Loyalität gewünscht. Der Innovations- und Gestaltungswille zeigt ebenfalls unterschiedliche Ausprägungen. Gemeinsam ist allen Ausprägungen, dass eine eigenverantwortliche und proaktive Arbeitsweise gewünscht wird, die sich durch kreative Ziel‑, Lösungs- und Serviceorientierung sowie Engagement auszeichnet. Auch sollen Bewerber:innen entscheidungsstark sein. Der Innovations- und Gestaltungswille im engeren Sinne gliedert sich in eine passive Ausprägung, die durch Offenheit und Bereitschaft gegenüber Innovation gekennzeichnet ist, und eine aktive Ausprägung, in der innovative Ideen gefordert werden und auch die Leitung der Umsetzung vorangetrieben werden soll. Das selbstsichere Auftreten und der offene Umgang mit Menschen erfordern ein ausgeprägtes Verhandlungsgeschick und -sicherheit sowie Konfliktfähigkeit und Belastbarkeit, aber auch das Attribut eines Teamplayers.

### Verantwortungen

**Abb. 3:**
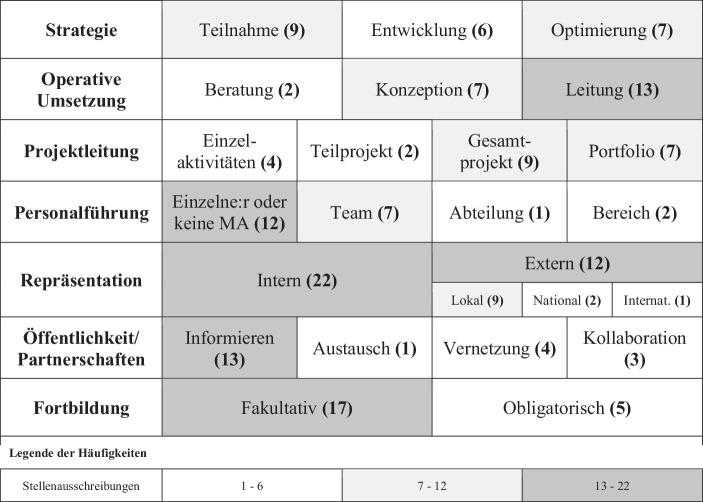
Axiale Kodierungsergebnisse der Verantwortungsausprägungen (Häufigkeiten in Klammern und farblich hervorgehoben)

Während einzelne Kompetenzen bereits mehrere Ausprägungen zeigten, so gilt dies durchgängig für die identifizierten Verantwortungen der IT-Führungsrollen. Abb. 3 zeigt die Verantwortungen mit deren Ausprägungen und Häufigkeiten.

Die Verantwortungen für Repräsentation, Öffentlichkeit und Partnerschaften sind hervorzuheben, neben den zu erwartenden Verantwortungen für Strategie, operative Umsetzung, Projektleitung, Personalführung und Fortbildung. Im öffentlichen Sektor umfasst die Repräsentation des Fachbereiches sowohl die Repräsentation innerhalb der Organisation als auch außerhalb der Organisation zur Beratung und Mitwirkung in der Fachpolitik (z. B. in Ministerien oder inter-/nationalen Gremien). Auch die Vernetzung mit anderen Organisationen (insb. Behörden) und Akteuren, insbesondere auf Kommunalebene, gehört zu den Verantwortungen von IT-Führungsrollen. Die Häufigkeitsverteilung der Ebenen der externen Repräsentation (kommunal, national, international) entspricht größtenteils der Datenmaterialverteilung auf die Ebenen im öffentlichen Dienst (Kommune, Land, Bund), einzig IT-Führungsrollen in öffentlichen Hochschulen und Forschungseinrichtungen sind nicht zuordnungsfähig. Die Häufigkeiten zeigen, dass IT-Führungsrollen im öffentlichen Sektor zu einer starken operativen Umsetzung bei einer schwächeren strategischen Verantwortung tendieren. Fortbildung wird nur in wenigen Fällen als obligatorisch angesehen.

### Aufgaben

Als rollenübergreifende Aufgaben wird das Management von Innovation, Veränderungen, Projekten, Prozessen, interner und externe Stakeholder genannt. Darüber hinaus gilt es für die IT-Führungsrollen Controlling, Monitoring und Qualitätssicherung der Maßnahmen sicherzustellen.

Die Chief Digital Officers analysieren und bewerten technologische Trends und steuern den digitalen Transformationsprozess der Organisation durch Digitalisierungs- und Innovationsprojekte. Dafür sollen auf Kommunalebene Fördermittel akquiriert und verwaltet werden. Inhaltlich werden als Projekte eGovernment, insb. E‑Akte, digitales Anordnungswesen und die Einführung eines Dokumentenmanagementsystems in den Stellenausschreibungen genannt. Auf der Kommunalebene liegt der Fokus auf der Erhöhung der Standortqualität durch Smart-City-Projekte. Dabei sollen Gesundheits- und Bildungswesen, Kommunikationsinfrastruktur, Verkehrssysteme und Energieanwendungen umweltgerecht, nachhaltig und digital vernetzt werden. Es wird dabei auf eine Partnerschaft von kommunalen und privatwirtschaftlichen Unternehmen, Behörden, Institutionen und Bildungseinrichtungen gesetzt. Vereinzelt werden neben Smart City auch Elektromobilität und Breitbandausbau als Projektthemen auf Kommunalebene genannt.

Die Chief Information Security Officers haben als primäre Aufgabe den Aufbau, die Weiterentwicklung und die Steuerung des Managementsystems für Informationssicherheit (ISMS) zu gewährleisten mit entsprechender Dokumentation der technischen und organisatorischen Maßnahmen. Daraus folgt die Einführung, Überwachung und strategische Weiterentwicklung von IT-Sicherheitsrichtlinien, -maßnahmen, -prozessen und -zielen. Die Informationssicherheit soll durch Begleitung von IT-Projekten, Audits und Revisionen sichergestellt werden. Oft werden auch Business Continuity und Incident Management als operativer Aufgabenbereich genannt. Der Entwurf und die Durchführung von Sensibilisierungsmaßnahmen und Schulung von Mitarbeitenden in der Organisation gehören zum Aufgabenbereich der CISOs ebenso wie Risikomanagement und die Bewertung technologischer Trends und Innovationsthemen mit Bezug zur IT-Sicherheit.

Die Chief Information Officers entwickeln und steuern eine auf die Organisationsstrategie abgestimmte IT-Strategie und -Governance. Außerdem sollen Prozesse digitalisiert und strategisch weiterentwickelt werden. Ressourcen und Budget sowie die Kernprozesse der IT, Betrieb, Entwicklung, Architektur und Infrastruktur, sollen gesteuert werden.

### Empirische Rollenverteilung der IT-Führungsrollen

**Abb. 4:**
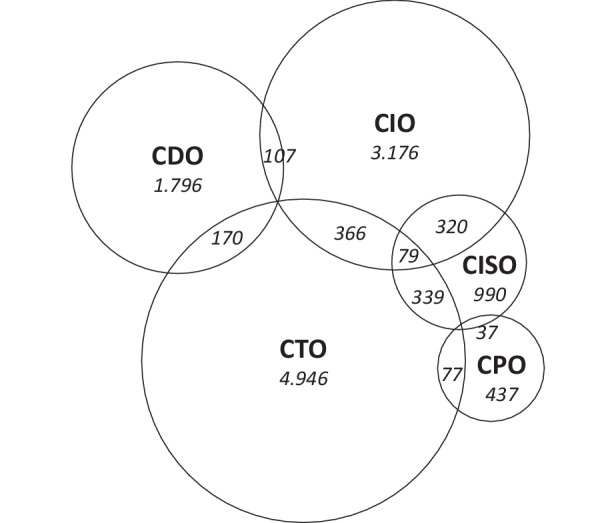
Venn-Diagramm zur empirischen Rollenverteilung der IT-Führungsrollen (*CIO* Chief Information Officer, *CDO* Chief Digital Officer, *CISO* Chief Information Security Officers, *CTO* Chief Technology Officer, *CPO* Chief Product Officer)

Neben der qualitativen Auswertung der Kompetenzen, Verantwortungen und Aufgaben wurden die Stellenausschreibungen auch quantitativ ausgewertet. Dafür wurden die Titel aller 12.934 Stellen in den IT-Fachbereichen auf Schnittmengen der Datenerhebungs-Filterung („digital, informati, produ[c,k]t, sicherheit, security, techno, IT-“) untersucht und als Venn-Diagramm in Abb. 4 dargestellt. So gibt es 170 IT-Stellen, die sowohl „digital“ (der CDO-Führungsrolle zugeordnet) als auch „IT-“ (CTO) im Titel enthalten. Die größte Gruppe der Stellen, die IT-Bindestrich-Positionen wie IT-Administration, IT-Applikationen oder IT-Infrastruktur, gehören zur CTO-Rolle, während der CIO-Rolle „informati*“ zugeordnet ist. In den ausgewerteten Stellenausschreibungen des TMT gab es keine CPO-Stellen, jedoch gibt es Fachstellen in diesem IT-Fachbereich.

## Diskussion der Auswahlkriterien zur Bestenauslese im öffentlichen Sektor

Verfassungsrechtlich ist vorgeschrieben, dass alle deutschen Bewerber:innen aufgrund ihrer „Eignung, Befähigung und fachlichen Leistung gleichen Zugang zu jedem öffentlichen Amte“ (Art. 33 Abs. 2 GG) haben und nach dem Grundsatz der Bestenauslese ausgewählt werden (BAG vom 10. Februar 2015 – 9 AZR 554/13). Diese Anforderung erstreckt sich in den meisten Bereichen auch auf nicht-deutsche Berwerber:innen. Die Bestenauslese, eine Qualitätssicherungsmaßnahme, dient dem öffentlichen Interesse (BVerwG 25. November 2004 – 2 C 17.03 – BVerwGE 122, 237). Daraus folgt für die Praxis, dass Stellenausschreibungen im öffentlichen Dienst auf einem verpflichtenden Anforderungsprofil basieren. Dieses definiert die im Auswahlverfahren nicht-veränderbaren Entscheidungskriterien (Wulfers 2017), so dass alle Bewerber:innen die gleichen Chancen im Auswahlverfahren haben (BAG 6. Mai 2014 – 9 AZR 724/12 – Rn. 10 mwN).

Im Folgenden werden die empirischen Anforderungsprofile der IT-Führungsrollen im öffentlichen Dienst kritisch auf ihre Eignung als nicht-veränderbare Entscheidungskriterien zur Sicherstellung der chancengleichen, aber auch realistischen Bestenauslese mit dem Ziel der „besten“ Stellenbesetzung evaluiert.

Die Anforderungsprofile erfordern i. d. R. nur ein abgeschlossenes Hochschulstudium, jedoch ohne Festlegung auf die Fachrichtung. Diese Aufweichung der Bestenauslese lässt sich mit dem hohen Quereinstieger:innen-Anteil in IT-Berufen in Einklang bringen. Nur ein Bruchteil der IT-Berufstätigen haben eine entsprechende formale Berufsausbildung bzw. ein entsprechendes Studium (Zehnder 2004), so dass die Aufweichung aus praktischen Gründen erfolgen muss, sofern die Stelle realistisch besetzt werden soll. Ungewöhnlich sind jedoch die (fast) nicht geforderten Englisch-Kenntnisse für die IT-Führungsrollen. Die englische Sprache ist die *lingua franca *in der IT, so dass diese in allen Anforderungsprofilen verpflichtend sein sollte. Auch die Abwesenheit von agilen Methoden ist verwunderlich, so werden diese doch häufig von IT-Führungskräften gefordert (Eberl und Drews 2021). Gleichzeitig definieren auch die geforderten Attribute Anforderungen an Bewerber:innen, wie die eigenverantwortliche und proaktive Arbeitsweise und Innovations- und Gestaltungswille, die auch im agilen Umfeld notwendig sind (Kusuma et al. 2019; Gerster et al. 2018). Andererseits sind die im öffentlichen Dienst bekannten Organisationsstrukturen und -methoden nicht an agile Prinzipien angelehnt, auch wenn es einige Veröffentlichungen für eine agile Verwaltung gibt (Bartonitz et al. 2018).

Obwohl dem lebenslangen Lernen ein großer Stellenwert in der Literatur zugewiesen wird (Kiefer et al. 2021; Kusuma et al. 2019), spiegelt sich dies nur eingeschränkt in den untersuchten Stellenausschreibungen wider. Die Bedeutung des lebenslangen Lernens in der IT, auch für Führungsrollen, wird noch deutlicher, sofern der Humankapitalwertverlust in Betracht gezogen wird. Die Wissensrelevanzzeit für IT-Kenntnisse beträgt lediglich ein Jahr (Scholz 2018), so dass das Humankapital jährlich einen hohen zweistelligen Wertverlust erleidet, sofern keine aktive Personalentwicklung betrieben wird. Insofern verwundert eine scheinbar nachrangige Bedeutung in den Auswahlkriterien zur Stellenbesetzung.

Die Verteilung der Verantwortungsausprägungen zeigen, dass die IT-Führungsrollen in Strategieprozessen nur teilnehmen oder entwickeln, jedoch selten optimieren. Für diese strategische Schwäche könnte der politischer Willensbildungsprozess eine Erklärung sein, der dem öffentlichen Dienst traditionell eher die Rolle der operativen Umsetzung zuweist. Dass die Organisation von Fortbildungen selten als Verantwortung gesehen wird, ist ein weiteres Indiz für die schwächere Bedeutung des lebenslangen Lernens; wie bereits oben ausgeführt. Die Aufgaben der CDO-Rollen sind i. d. R. mit konkreten Projekten inhaltlich beschrieben. Auch die CISO-Rollen haben eine ausführliche Beschreibung, die sich an den BSI-Standards orientieren. Lediglich die CIO-Rollen basieren auf einer Sammlung von allgemeinen IT-Managementprozessen ohne weiterführende Beschreibung.

Für die Auswahlkriterien der IT-Führungsrollen können einige bedeutsame Handlungsempfehlungen ausgesprochen werden. Für die Kompetenzen empfehlen wir höhere Englisch-Kenntnisse mit aufzunehmen, sofern noch nicht vorhanden, sowie stärker ausgeprägte IT- und Digitalkenntnisse für IT-Führungsrollen. Aufgrund der hohen Humankapital-Wertverluste in der IT sollte auch ein höherer Fokus auf lebenslanges Lernen gesetzt werden. Agile Methoden sollten als erforderliche Kompetenzen ebenfalls aufgenommen werden. Vorsicht ist auch bei der Überbetonung von Verwaltungskenntnissen geboten, die Bewerber:innen außerhalb der Verwaltung abschreckt.

## Interpretation und Forschungsausblick

**Abb. 5:**
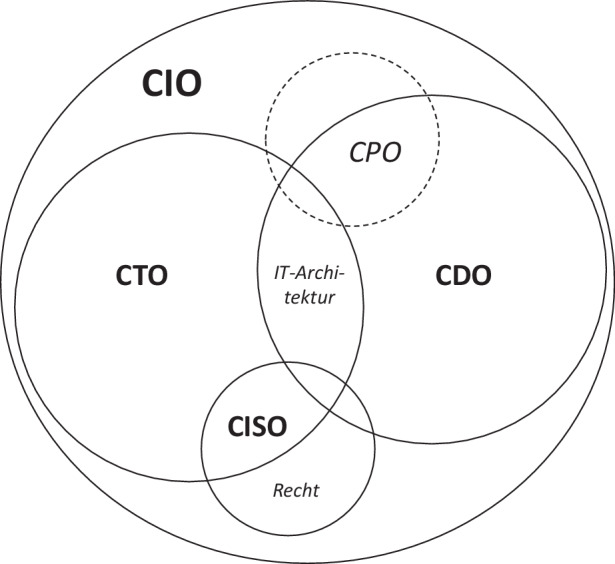
Venn-Diagramm zur hypothetischen Rollenverteilung der IT-Führungsrollen

Ausgehend von der qualitativen Auswertung der Kompetenzen, Verantwortungen und Aufgaben der IT-Führungsrollen im TMT sowie der empirischen Rollenverteilung in Abb. 4 schlagen wir die hypothetische Rollenverteilung in Abb. 5 vor. Dabei soll aufgezeigt werden, wie Zusammenspiel und Abgrenzung durch verschiedene Personen besetzter IT-Führungsrollen ausgestaltet werden könnte. Statt einer CDO-CIO-Dyade, wie sie in der Literatur oft vorgeschlagen wird (Horlacher 2016; Horlacher und Hess 2016), ist in der Praxis des öffentlichen Sektors eine CTO–CDO-Dyade mit einer übergeordneten CIO-Rolle und untergeordneten spezialisierten CPO- und CISO-Rollen vermutlich zielführender. Die IT-Führungsrollen treffen sich alle im Verantwortungs- und Aufgabenbereich IT-Architektur und haben je nach Rolle weitere Überschneidungen. Ähnliche Schnittmengen-Modelle sind bereits für CIO und CDO in der Literatur zu finden (Walchshofer und Riedl 2017), jedoch fehlt bisher ein ganzheitlicher Blick auf alle IT-Führungsrollen. Die CTO-Rolle ist nicht in den erhobenen Daten enthalten, jedoch wird eine vergleichbare Technologie-fokussierte Führungsrolle, die eine Unterabteilung leitet und insofern in den Suchkriterien ausgeschlossen wurde, im Datenmaterial mehrfach erwähnt und auch die Behördenorganigramme der ausgewerteten Stellen zeigen zum Teil die drei Rollen CTO, CDO und CIO.

Ein mögliches Gegenargument gegen eine Ausgestaltung einer CTO–CDO-Dyade ist der geringe Transformationsanspruch der CIO-Rolle, während dies Kernbestandteil der CDO-Rolle ist. Auch findet sich im Datenmaterial keine CPO-Rolle, jedoch sollte dies als eine Besonderheit des öffentlichen Dienstes interpretiert werden. Die CPO-Rolle im Sinne eines agilen interdisziplinären Product Owners wie bei SCRUM, die als Führungsrolle im TMT ein strategisches Produktportfolio verantwortet, ist mit klassischen Organisationsstrukturen des öffentlichen Sektors und der daraus folgenden strikten Aufteilung von Zuständigkeiten und strikten Hierarchien schwer vereinbar. Die CPO-Rolle konzentriert sich im Gegensatz zum projekt- und transformationsgetriebenen CDO auf sozio-technische Informationssysteme als IT-Produkte, auch wenn sich Projekte und Produkte gegenseitig beeinflussen und insofern die beiden Rollen stark verbunden sind. Im öffentlichen Dienst scheint der Fokus für Product Owner in der IT-Sicherheit und im IT-Betrieb zu liegen. Die CISO-Rolle ist stark technologisch geprägt und wird daher näher zum CTO eingeordnet und grenzt sich durch die ausgeprägten rechtlichen Kompetenzen von den anderen IT-Führungsrollen ab. Die übergeordnete Funktion der CIO-Rolle wird auch anhand der überdurchschnittlichen Vergütung (E14/15 bzw. als einzige Stelle auch außertariflich) sowie des durch IT-Strategie und -Governance geprägten Aufgabenprofiles in den empirischen Daten betont. Exemplarisch soll die Stellenausschreibung der CISO-Stelle der Hochschule Ruhr West für die vorgeschlagene hypothetische Rollenverteilung zitiert werden: *„Dazu tauschst du dich regelmäßig mit dem CIO und den Kolleg:innen in den Bereichen Digitalisierung *[CDO]* und IT *[CTO]* aus.“*

Zukünftige Forschung sollte die CTO–CDO-Dyade empirisch weiter untersuchen, ebenso wie die Schnittmengen zwischen den IT-Führungsrollen. Die empirischen Ergebnisse zeigen zwar eine erste empirische Evidenz für eine mögliche CTO–CDO-Dyade, jedoch sollte diese ausgebaut werden, bevor Handlungsempfehlungen entwickelt werden. Dazu könnte eine weiterführende Interviewstudie mit den nun in der Zwischenzeit besetzten Stellen als Interviewpartner:innen in Fragen kommen oder eine qualitative Auswertung der Stellenausschreibungen der darunterliegenden IT-Führungsrollen. Auch die Analyse von Organigrammen wäre denkbar.

## Fazit und Limitationen

Die vorliegende Studie zeigt, dass Job-Mining eine geeignete Methode zur Datenerhebung ist und sich gut mit einer sich anschließenden qualitative Inhaltsanalyse kombinieren lässt. Die Interpretationskraft ist für den öffentlichen Sektor aufgrund der Verbindlichkeit der ausgeschriebenen Anforderungsprofile gesteigert. Inhaltlich überrascht, dass IT-Führungsrollen im öffentlichen Sektor auch eine externe Repräsentationsverantwortung beinhalten und die Verantwortungen breite Spannweiten besitzen. Es wurden an die Praxis im öffentlichen Dienst gerichteten Handlungsempfehlungen zur Anpassung der Auswahlkriterien formuliert und ein Forschungsausblick für IT-Führungsrollen und einer möglichen CTO–CDO-Dyade gegeben. Limitationen sind die empirische Fallzahl und insbesondere die Abwesenheit weiterer Rollenprofile neben den detailliert untersuchten CIO-, CDO- und CISO-Rollen. Eine Vertiefung der bisherigen Forschung, z. B. durch Interviews der nun besetzten Stellen, lässt weitere Erkenntnisse erwarten.

## Footnotes

1 CPO (Chief Product Officer) verantworten die strategische Ausrichtung der digitalen Produkte in einer Organisation und sind die TMT-Führungskraft für die Product Owner im Sinne der agilen Methode Scrum.
